# Full-Length Laminin-332 Promotes Integrin α3β1-Dependent Cutaneous Wound Closure in a Murine Model

**DOI:** 10.3390/biomedicines14091969

**Published:** 2026-09-01

**Authors:** Pratik Vangal, Mia Cortese, Salima Lalani, Ramon Bossardi Ramos, Whitney M. Longmate

**Affiliations:** 1 Department of Molecular and Cellular Physiology, Albany Medical College, Mail Code 8, Room MR-424, 47 New Scotland Avenue, Albany, NY 12208, USA; 2 Department of Surgery, Albany Medical College, Albany, NY 12208, USA

**Keywords:** integrin, laminin, extracellular matrix, re-epithelialization, keratinocyte, wound healing, nerve growth factor

## Abstract

**Background:** Cutaneous wound repair requires rapid re-epithelialization to restore barrier function and protect against infection. This process depends on coordinated keratinocyte migration and proliferation, and its impairment contributes to slow wound healing and increased healthcare burden. Current extracellular matrix (ECM)-based therapies largely target the dermis, while the therapeutic potential of epidermal basement membrane (BM) components remains underexplored. BM protein laminin-332 (LN332) is upregulated after injury and promotes keratinocyte migration. It functions as a key ligand for integrin α3β1, which is highly expressed by basal keratinocytes and further induced after wounding. **Objectives:** We aimed to investigate whether topical application of recombinant full-length LN332 could enhance wound repair in vivo and we examined the requirement for integrin α3β1 in this process. **Methods:** We used a full-thickness murine wound model with topical treatments of LN332 or a vehicle control, combined with a murine model of epidermis-specific integrin α3β1 deletion. **Results:** LN332 treatment accelerates re-epithelialization, and α3β1 is required for this effect. In addition to its direct effects on re-epithelialization, exogenous LN332 modulates several α3β1-dependent genes, including nerve growth factor (NGF). Our current study identifies NGF as an α3β1-dependent and LN332-responsive mediator of keratinocyte migration during wound healing. **Conclusions:** These findings identify the LN332–integrin α3β1-NGF axis as a promising therapeutic target for promoting re-epithelialization and improving healing outcomes.

## 1. Introduction

Following skin injury, epidermal repair is required to restore the skin barrier and protect against infection. In the process of wound re-epithelialization, wound edge keratinocytes proliferate and migrate across the wound bed to facilitate closure. Slow or faulty re-epithelialization increases the risk of infection and chronic wound formation. Clinically, the timely development of improved wound healing therapies is critical, as morbidity and healthcare costs associated with delays in wound healing impose a growing financial and social burden worldwide [1,2,3,4].

Current extracellular matrix (ECM)-based wound therapies focus on the addition of dermal collagen, mainly collagen I, to help restore the dermal matrix [5]. Meanwhile, ECM proteins found in the epidermal basement membrane (BM) have been largely overlooked. Laminin-332 (LN332), a major component of the epidermal BM, comprises the laminin α3, β3 and γ2 subunits. LN332 is upregulated within hours of wounding, deposited by keratinocytes into the provisional matrix, and promotes keratinocyte migration in vitro [6,7]. The full-length, unprocessed laminin γ2 arm of LN332 is required for its stable incorporation into the nascent BM [8].

LN332 is also the major ligand in skin for epidermal integrin α3β1 [9]. Integrins are heterodimeric transmembrane receptors consisting of an α and a β subunit, and are the major receptors for cell adhesion and migration [10]. Integrins are activated through engagement with ECM ligands, and keratinocytes express multiple integrins capable of binding ECM components within the provisional wound matrix [11,12]. Among these, integrin α3β1 is abundantly expressed by basal keratinocytes in the epidermis, is further upregulated following injury, and its functions during wound healing are thought to be largely conserved between rodents and humans [11,13,14,15].

Integrin α3β1 has been shown to mediate keratinocyte migration and polarization in vitro [14,15,16]. Indeed, it was previously observed in in vitro scratch wound assays that mouse keratinocytes expressing α3β1 (MKα3+) display organized, sheet-like migration while mouse keratinocytes lacking integrin α3β1 (MKα3−) fail to display coordinated migration and instead scatter into the open area of the wound [14]. Furthermore, a recent study from our group has shown a requirement for integrin α3β1 in efficient healing of wound epidermis in vivo [17]. In a murine wound model, we showed that induced epidermal deletion of *Itga3*, the gene that encodes the α3 integrin subunit, resulted in reduced wound closure with concomitantly reduced wound keratinocyte proliferation, indicating a critical role for integrin α3β1 in promoting wound re-epithelialization [17].

Since integrin α3β1 promotes cutaneous wound closure, the aim of our study was to determine whether LN332 treatment hastens re-epithelialization, and whether the presence of the integrin α3β1 receptor is required for this effect. Human recombinant, full-length LN332 is commercially available, and its recommended use is to coat culture dishes to support cell growth. Using a full-thickness murine wound model, in combination with inducible *Itga3* knockout, our current study demonstrates that LN332 supports wound re-epithelialization in vivo, and that integrin α3β1 is required for this effect. Our previous work has also established α3β1 as a major regulator of the keratinocyte transcriptome [18,19]. While it is likely that the α3β1-LN332 interaction stimulates keratinocyte migration directly, we also uncovered α3β1-dependent, LN332-responsive gene expression changes that contribute further to this healing effect. For example, nerve growth factor (NGF) has been shown previously to promote wound healing through stimulating epithelial migration [20,21]. Here, we validate keratinocyte-derived NGF as an α3β1-dependent, LN332-responsive factor both in vitro and in vivo, and we confirm NGF as a pro-migratory factor in keratinocytes. These findings demonstrate that the addition of exogenous non-fibrillar ECM components, such as LN332, may be a relevant biomaterial to develop for the wound clinic.

## 2. Materials and Methods

### 2.1. Murine Wound Healing Studies

K14^CreERT^::α3^flx/flx^ mice and wound healing protocols performed in this model were previously described [17,22]. All mice used in these studies were healthy and between 8 and 16 weeks of age. For integrin α3 epidermal knockout (α3eKO) experiments, five and three days prior to wounding, 1 mg of 4-hydroxytamoxifen in 200 μL acetone (4OHT; Caymen Chemical Company, Ann Arbor, MI, USA; iα3eKO) or acetone alone (vehicle control) was applied topically to the backs of shaved mice. For studies in integrin α3β1-expressing control mice, no pretreatment was performed. For the wounding procedure, mice were anesthetized, dorsum shaved, and two full-thickness wounds were created on the back of each mouse using a sterile 4 mm biopsy punch (Integra, Princeton/Plainsboro, NJ, USA), as described [23]. Each wound was topically treated daily for three consecutive days beginning on the day of wounding with 35 μL of 0.1 mg/mL human recombinant full-length LN332 (BioLamina, Sundbyberg, Sweden) or with 35 μL of vehicle (PBS/10% glycine/0.02 NaN_3_) as control. For collagen experiments, wounds were treated with 35 μL of 3.75 mg/mL or 0.1 mg/mL rat tail collagen I (Corning, Corning, NY, USA) or with equal volume of vehicle (0.02 N acetic acid) as control. Wounds were permitted to heal for three days, after which time mice were euthanized by CO_2_ narcosis. Surgically excised wounds were bisected and embedded in OCT (Electron Microscopy Sciences, Morgantown, PA, USA) before freezing. To minimize potential confounding variables, experiments were performed using age-matched animals maintained under the same housing and environmental conditions. Animals were randomly assigned to experimental/treatment groups, and male and female mice were randomized independently in approximately equal proportions. All studies were institutionally approved and conducted in accordance with the NIH Guide for the Care and Use of Laboratory Animals.

### 2.2. Histology

Frozen sections were prepared as described [17]. Wound sections of 10 µm were rehydrated in 0.02% Tween-20/PBS for 10 min, fixed in 4% paraformaldehyde/PBS for 5–30 min, permeabilized in 0.4% Triton X-100/PBS for 5–30 min, blocked first in 3% BSA and 0.1% glycine in 0.1% Tween-20 for 30 min followed by a second block in 0.5% BSA, 10% goat serum, and 0.1% Tween-20 for 30–60 min, then stained with anti-α3 integrin subunit (1:750) [24], anti-K14 (1:1000; Biolegend, San Diego, CA, USA), or anti-Ki67 (1:200; Biolegend). Secondary antibodies (1:250; Molecular Probes, Eugene, OR, USA) were Alexa Fluor 488 goat anti-rabbit IgG or Alexa Fluor 594 goat anti-rat IgG. Sections were co-stained with DAPI to mark nuclei. Sections were mounted with ProLong Gold antifade mounting media (Molecular Probes).

### 2.3. Imaging and Quantitation

Micrographs were acquired using a Nikon (Tokyo, Japan) Eclipse 80i microscope equipped with either a Photometrics (Tucson, AZ, USA) Cool Snap ES camera or a Spot camera (Diagnostic Instruments, Sterling Heights, MI, USA). Image quantification was performed using NIS-Elements AR 3.2 or Fiji ImageJ 1.54p software. Wound gap length was determined by measuring the linear distance between opposing migrating epidermal wound edges, as previously described [17]. To quantify epidermal Ki67 positivity, the wound-proximal K14-positive epidermis, encompassing both follicular and interfollicular epidermis, was defined as the region of interest (ROI). Ki67-positive cells were counted within the K14-positive ROI, while total epidermal cell numbers were determined by counting all DAPI-positive nuclei within the same ROI. The percentage of Ki67-positive epidermal cells was calculated by dividing the number of Ki67-positive cells by the total number of epidermal cells and multiplying by 100, as previously described [17]. All imaging and quantitation were performed in a manner blinded to the experimental group.

### 2.4. Public Dataset Mining

A publicly available dataset (GSE28914; intact human skin versus wounded human skin) [25] was interrogated for differential gene expression (DGE) of the following gene panel: *ITGA3*, *ITGB1*, *LAMA3*, *LAMB3*, and *LAMC2*. A unified bioinformatics pipeline was developed using Python (v3). The upstream differential expression (DE) profiles, including log2 fold-changes and adjusted *p*-values, were derived from the National Center for Biotechnology Information (NCBI, Bethesda, MD, USA) Gene Expression Omnibus (GEO) data repository using the standard Web-based GEO2R pipeline (R 4.2.2, Biobase 2.58.0, GEOquery 2.66.0, limma 3.54.0). Significance was defined using Benjamini–Hochberg False Discovery Rate (FDR) adjusted *p*-values: **, *p* < 0.01; ***, *p* < 0.001. Heatmaps were rendered with Seaborn and Matplotlib using a Blue–White–Red colormap topology centered symmetrically at zero.

### 2.5. Cell Culture

Immortalized mouse keratinocytes lacking α3 integrin (MKα3−) and MK cells expressing α3 integrin (MKα3+; MK−/− cells reconstituted with human α3 integrin) have been previously described [26,27]. MK cells were cultured on collagen-coated dishes in supplemented low-calcium Minimum Essential Medium (MEM; Thermo Fisher Scientific, Waltham, MA, USA) and maintained at 33 °C in an atmosphere containing 8% CO_2_, as previously described [26,28]. For in vitro LN332 treatments, plated MKα3− and MKα3+ cells were incubated in MK culture medium supplemented with 0.02 mg/mL LN332 (BioLamina) or an equal volume of vehicle control (PBS/10% Glycine/0.02 NaN_3_) for 16–24 h, then processed for mRNA or protein analysis.

### 2.6. Immunoblotting

Whole-cell lysates were collected using a diluted 5× passive lysis buffer (Promega, Madison, WI, USA) with phosphatase and protease inhibitors (Roche, Basel, Switzerland) for protein detection. Immunoblotting was performed utilizing the following primary antibodies: anti-α3 integrin [24] (1:1000), anti-NGF (1:500; Abcam, Cambridge, UK, cat. # ab52918), anti-phospho-Erk1/2 (1:1000; Cell Signaling Technology, Danvers, MA, USA; cat. # 9101), anti-GAPDH (1:2000; Thermo Fisher Scientific; cat. # AM4300), or anti-ERK2 (1:1000; Santa Cruz Biotechnology, Dallas, TX, USA; cat. # sc-1647). Secondary antibodies used were HRP-conjugated donkey anti-mouse IgG (1:2000; Thermo Fisher Scientific; cat. # SA1-100) or donkey anti-rabbit IgG (1:2000; Thermo Fisher Scientific; cat. # SA1-200). Chemiluminescent signals were detected using a Bio-Rad ChemiDoc MP imaging system with Image Lab 6.0.1 build 34 software (Bio-Rad, Hercules, CA, USA). Band intensities were quantified using the “Volume Tools” function and normalized to the corresponding loading control.

### 2.7. siRNA Transfection

MKα3+ cells were seeded on collagen-coated 6-well dishes in full medium 24 h prior to transfection. Transfection was performed using Lipofectamine RNAiMAX (Life Technologies, Carlsbad, CA, USA). Two siRNAs targeting *Ngf* (20 nM; Integrated DNA Technologies, Coralville, IA, USA) were used: siRNA#1 5′-UGUUCUACACUCUGAUCACUGCGTT-3′ and siRNA#2 5′-GUCAAGGCGUUGACAACAGAUGAGA-3′, and a non-targeting siRNA was used as control. After 72 h, confluent cultures were used in downstream scratch wound migration experiments and then RNA was isolated to assess knockdown by RT-qPCR.

### 2.8. In Vitro Scratch Wound Migration Assays

MK cells (either native or siRNA transfected, depending on the experiment) were grown to confluence on collagen-coated tissue culture dishes. Cultures were serum-starved overnight, scratch wounded with the narrow end of a 200 μL pipette tip, then rinsed several times with PBS to dislodge remaining cells. Phase images were collected just after scraping (T_0_) on a Nikon Eclipse 80i using a Spot camera (Diagnostic Instruments). For LN332 scratch wound studies, culture medium was replaced and supplemented with either 0.02 mg/mL LN332 (BioLamina) or an equal volume of vehicle control (PBS/10% Glycine/0.02 NaN_3_). For *Ngf* knockdown scratch wound studies, cultures were replaced with full culture medium absent LN332 to assess the role of NGF independently. Cultures were replaced into culture incubator, then phase images were collected at a single final timepoint 18 h (T_18_) or 24 h (T_24_) post-scraping, depending on the experiment. NIS Elements AR 3.2 software was used for quantitation. First, wound area was determined at each timepoint (T_0_ and T_18/24_) by drawing a polygon to outline the “open” wound and using the area tool. Percent wound closure was then calculated with the following equation: ((Area T_0_ − Area T_18/24_)/Area T_0_) × 100. Relative wound closure was calculated when data were normalized by the daily average to account for variability by day. Quantitation was performed in a manner blinded to experimental group.

### 2.9. Trametinib Treatment of MK Cultures

MKα3+ cells were treated with 200 nM of a Mitogen-Activated Protein Kinase (MEK) inhibitor, trametinib (Cayman Chemical, Ann Arbor, MI, USA; cat. # 16292), for 72 h in MK culture medium, or left untreated as a control. Protein was isolated for immunoblotting, as described above, and MEK inhibition was confirmed with phospho-ERK immunoblots.

### 2.10. RNA Sequencing

Trizol-based RNA extraction and RNA sequencing of MKα3− and MKα3+ cells with either vehicle or laminin treatment (at 0.02 mg/mL as described above) were conducted by Genewiz (Azenta Life Sciences, Burlington, MA, USA; data available at the GEO repository, accession number GSE336489). Downstream analyses were performed by the Bioinformatics Core at Albany Medical College. High-throughput sequencing was performed on an Illumina (San Diego, CA, USA) NovaSeq X Plus system. Sequencing reads were mapped to the mouse reference genome mm10 using the align function from Rsubread v2.10.5 with default settings [29]. Gene-level read counts were obtained with featureCounts using the built-in Entrez gene annotation, and gene symbols were assigned based on NCBI annotation. Lowly expressed genes were removed by retaining genes with counts per million (CPM) greater than 0.5 in at least three samples. Raw counts were converted to log2-CPM values and normalized by the trimmed mean of M-values method implemented in edgeR 4.10.0 [30]. Differential gene expression was performed using the limma-voom workflow, followed by empirical Bayes moderation [31]. Genes were considered differentially expressed when they met an adjusted *p*-value threshold of <0.05 and an absolute log2 fold-change > 0.5.

### 2.11. Isolation of RNA and RT-qPCR

The RNeasy Plus Mini Kit (Qiagen, Hilden/Duesseldorf, Germany) was used to isolate total RNA from MK cells, then cDNA was synthesized using iScript Reverse Transcription Supermix (Bio-Rad). RT-qPCR was performed with SsoAdvanced Universal SYBR Green Supermix (Bio-Rad) on a Bio-Rad CFX96 Touch thermocycler with CFX Maestro 1.1 (v 4.1.2433.1219) software with the following conditions: 95 °C for 2 min (1 cycle); 95 °C for 5 s and 60 °C for 30 s (39 cycles); melt curve analysis from 65 to 95 °C, with 0.5 °C increments every 5 s. Primer sequences for *Ngf* were as follows: 5′-CAGTGAGGTGCATAGCGTAAT-3′ and reverse 5′-CTCCTTCTGGGACATTGCTATC-3′. The geometric mean of three reference genes (*Ppia*, *Tbp*, and *Nono*; Bio-Rad) was used for normalization and relative mRNA levels were calculated using the 2^−(ΔΔ*C*_t_)^ method [32].

### 2.12. RNA In Situ Hybridization (ISH)

Ten µm-thick tissue cryosections were fixed in 4% PFA for 1 h, dehydrated through a graded ethanol series (50%, 70%, and 100%; 5 min each), treated with hydrogen peroxide for 10 min, and subsequently permeabilized with Protease IV for 20 min. RNA ISH was performed using the RNAscope Fluorescent Multiplex V2 kit from Advanced Cell Diagnostics (a Bio-Techne brand, Singapore; cat. #323110). Sections were incubated for 2 h at 40 °C with probes targeting *Ngf* mRNA (cat. #446331-C3) or *Krt14* mRNA (cat. #422521-C1), the latter used to identify keratinocytes. Positive technical controls targeting murine housekeeping genes and negative controls targeting bacterial genes were included in all experiments (Advanced Cell Diagnostics, Newark, CA, USA). Signal amplification and horseradish peroxidase (HRP) development were performed according to the manufacturer’s instructions. Sections were counterstained with DAPI to visualize nuclei and mounted with ProLong Gold Antifade Reagent (Thermo Fisher Scientific). Images were acquired as described above, and mRNA puncta were quantified using Fiji ImageJ 1.54p (NIH) with the “Analyze Particles” function. Total *Ngf* mRNA signal was normalized to cell number within the selected wound ROI. Quantitation was performed in a manner blinded to the experimental group.

### 2.13. Statistical Analysis

Data are presented as mean ± standard error of the mean (SEM). For in vivo studies, data points represent a wound from an animal. Since each wound is independently created, treated, and analyzed, the wound is considered the experimental unit, as accepted for murine excisional wound healing studies [33]. For wound studies, each test group consisted of at least 6 mice with two wounds each, for a total of at least 12 wound samples, which provides adequate power (>80%) to detect changes larger than one standard deviation, *p* < 0.05 based on published murine wound studies analyzing epidermal phenotypes in integrin-deficient mice [23,34,35,36]. Throughout these studies, two wounds in total were removed due to inclusion of bedding material into the wound that may confound healing parameters. Normality of the data was established by the Shapiro–Wilk normality test. For in vitro studies, data points represent experimental replicates. Experimental groups were compared using Student’s *t*-test or one-way ANOVA with multiple comparisons, as appropriate. All data analyses, including normality tests, were performed using GraphPad Prism version 9.0 for Windows.

## 3. Results

### 3.1. Topical Treatment with LN332 Stimulates in Vivo Murine Wound Closure in an Integrin α3β1-Dependent Manner

**Figure 1:**
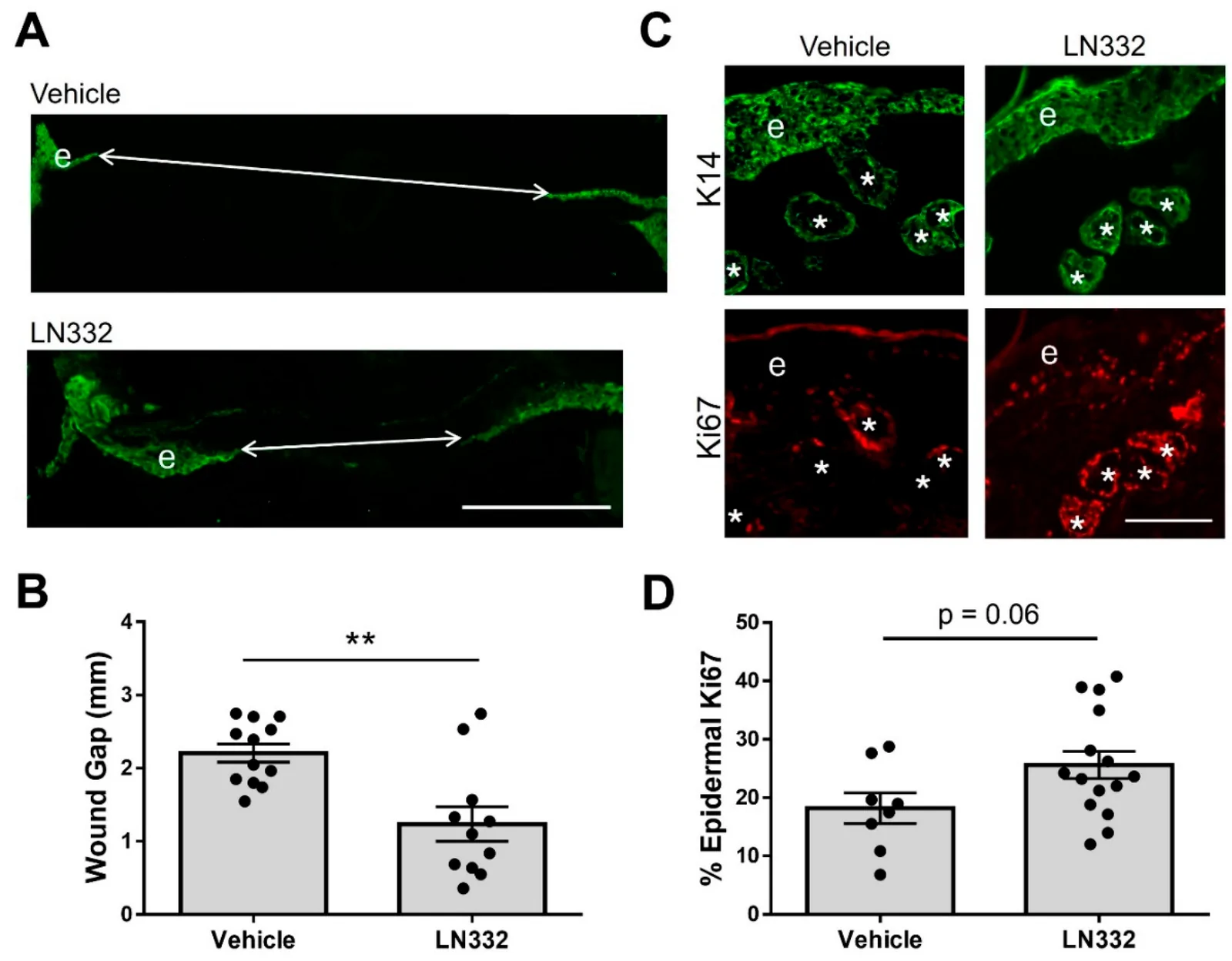
LN332 treatment accelerates cutaneous murine wound closure. Full-thickness wounds on mouse dorsum were treated for three consecutive days with LN332 or vehicle control. (**A**,**C**) Cryosections of 3-day, partially re-epithelialized wounds were prepared. Immunofluorescence with (**A**) anti-K14 to mark the epidermis (green) or (**C**) anti-K14 (green) together with proliferation marker anti-Ki-67 (red); representative images shown; (**A**) arrows indicate wound gap; e, epidermis; scale bar = 500 μm; (**C**) e, epidermis; *, hair follicle; scale bar = 50 μm. (**B**) Graph shows wound gap distance between wound edges as shown in (**A**). (**D**) Graph shows percentage of epidermal cells that are Ki67-positive as shown in (**C**). Data are mean ± SEM; *n* ≥ 8 wounds per treatment group; Student’s *t*-test; **, *p* < 0.01; *p* value approaching significance reported.

**Figure 2:**
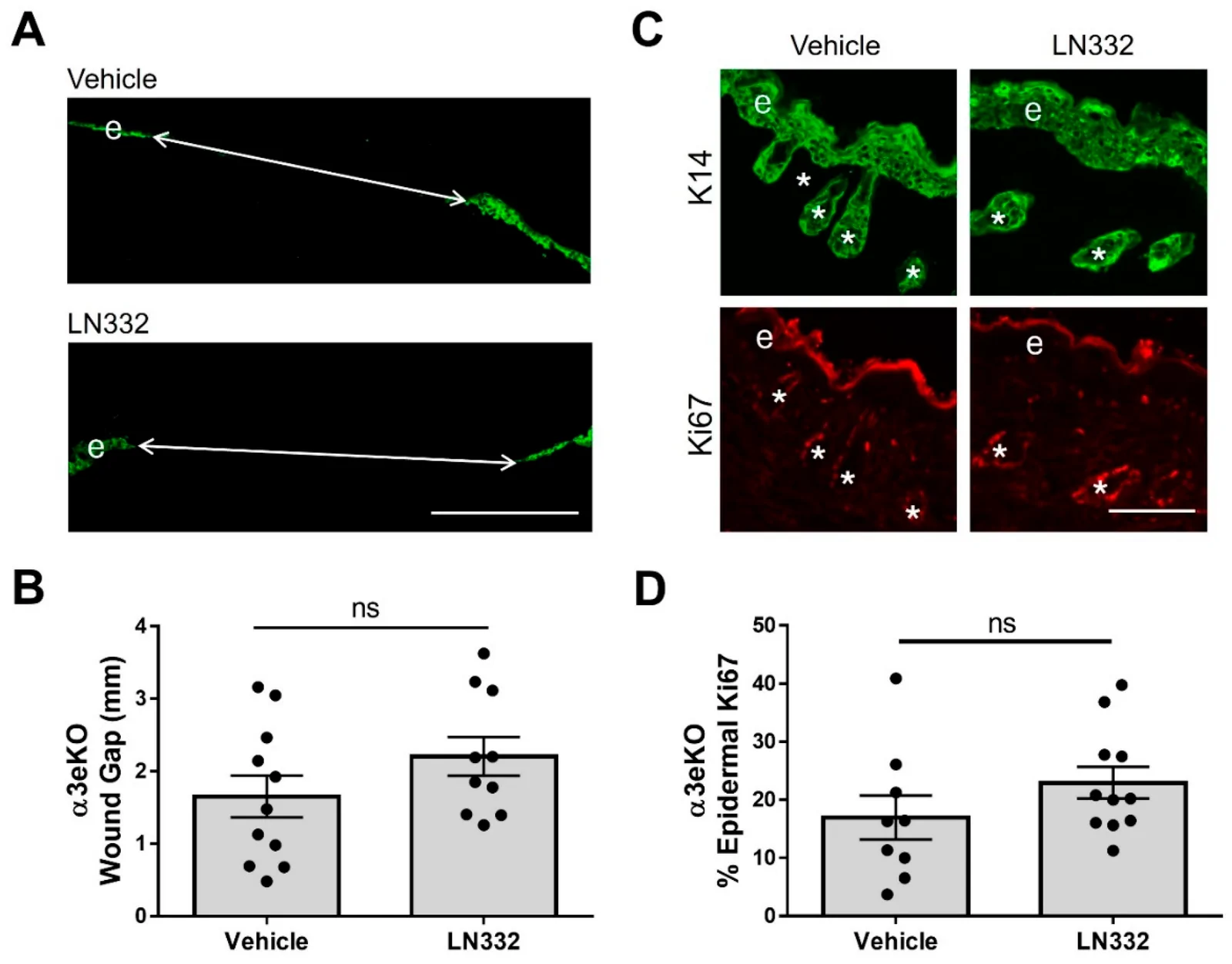
LN332 treatment does not accelerate cutaneous wound closure in mice that lack epidermal LN332 receptor, integrin α3β1. K14^CreERT^::α3^flx/flx^ mice were treated with topical 4OHT five and three days prior to wounding to knockout epidermal integrin α3β1. Full-thickness wounds on mouse dorsum were treated for three consecutive days with LN332 or vehicle control. (**A**,**C**) Cryosections of 3-day, partially re-epithelialized wounds were prepared. Immunofluorescence with (**A**) anti-K14 to mark the epidermis (green) and (**C**) anti-K14 (green) together with proliferation marker anti-Ki-67 (red); representative images shown; (**A**) arrows indicate wound gap; e, epidermis; scale bar = 500 μm; (**C**) e, epidermis; *, hair follicle; scale bar = 50 μm. (**B**) Graph shows wound gap distance between wound edges as shown in (**A**). (**D**) Graph shows epidermal cell percentage that is Ki67-positive as shown in (**B**). Data are mean ± SEM; *n* ≥ 9 wounds per treatment group; Student’s *t*-test; ns, not significant.

To test the efficacy of topical LN332 treatment in cutaneous wound healing, and to assess the requirement of its receptor integrin α3β1 in this process, we used young adult K14Cre-ERT::α3flx/flx mice [22] with epidermal α3β1 expression intact (control mice; Figure 1) or with tamoxifen-induced deletion of epidermal α3β1 (α3eKO mice; Figure 2). Immunofluorescence staining revealed efficient deletion of α3β1 from the epidermis of tamoxifen-treated α3eKO skin compared to controls (Figure S1).

It is worth noting that “wound closure” generally refers to the overall restoration of tissue integrity, while “re-epithelialization” is the primary regenerative step in the process of wound closure where the epidermis is restored. Our studies assess the success of re-epithelialization, a critical component of wound closure, over a fixed period of time by measuring the “wound gap,” or the unclosed length between the two edges of a wound. For example, the smaller the wound gap remaining, the further along re-epithelialization has progressed, which is indicative of improved wound closure.

First, full-thickness 4 mm punch wounds were made on the backs of integrin α3β1-expressing control mice, and the wounds were topically treated daily with recombinant LN332 or with vehicle as a control for three consecutive days as they healed. Wound gap measurements revealed that LN332-treated wounds showed significantly increased wound closure (i.e., smaller wound gaps) compared with vehicle-treated wounds, indicating enhanced re-epithelialization (Figure 1A,B).

The re-epithelialization process involves the coordinated migration of keratinocytes across the wound bed. Additionally, the proliferation of wound edge keratinocytes, which mainly occurs in the stem compartments within hair follicle bulges, increases the number of keratinocytes available to migrate, further promoting re-epithelialization [37]. Ki67 immunofluorescence suggested a trend toward increased wound-proximal epidermal proliferation in LN332-treated wounds compared to vehicle-treated wounds, although it did not reach statistical significance (*p* = 0.06) (Figure 1C,D). Together, these data indicate that wound closure enhancement seen in the LN332-treated wounds may be primarily attributed to increased keratinocyte migration, rather than proliferation.

Next, the same experiment was performed on α3eKO mice which lack expression of epidermal integrin α3β1, a major receptor in skin for LN332. In wounds of α3eKO mice, no significant difference was observed in wound gap size (Figure 2A,B) or in wound-proximal epidermal proliferation (Figure 2C,D) between vehicle- and LN332-treated wounds. This indicates that expression of integrin α3β1 is required for the pro-healing effect of LN332 wound treatment that was observed in control mice (Figure 1).

Interestingly, interrogation of a publicly available dataset (GSE28914) from human tissue revealed that mRNA levels of all subunits of both integrin α3β1 and LN332 (i.e., *ITGA3*, *ITGB1*, *LAMA3*, *LAMB3*, and *LAMC2*) are upregulated in healing wounds compared to intact skin (Figure S2), indicating that the α3β1-LN332 axis is relevant to human cutaneous wound healing.

Collagen is an ECM component that is commonly used as an addition to wound dressings to support healing [38]. Therefore, we tested the effect of topically applied collagen I on wound gap size of control mice wounds, as was done for LN332 (Figure 1). When used at the same concentration as LN332 (0.01 mg/mL), collagen treatment did not change wound gap size compared to vehicle control (Figure S3). However, a trend towards a healing benefit was observed with high concentration (3.75 mg/mL) collagen treatment, as indicated by reduced wound gap size (*p* = 0.074), compared to vehicle control (Figure S3).

### 3.2. RNA Sequencing of Keratinocytes Identifies Gene Expression Changes That Are α3β1-Dependent and LN332-Responsive

**Figure 3:**
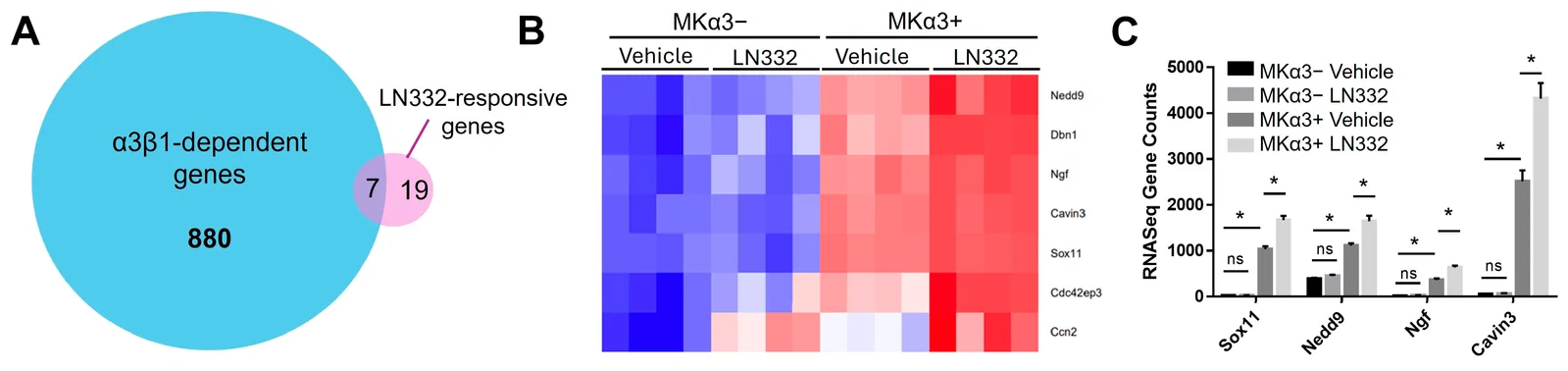
RNA sequencing identifies α3β1-dependent and LN332-responsive genes of the keratinocyte transcriptome. RNA from MKα3− and MKα3+ cells treated with LN332 or vehicle control were submitted for RNA sequencing (Genewiz). (**A**) Venn diagram depicting the number of α3β1-dependent (blue circle; 880 genes) and LN332-responsive genes (purple circle; 19 genes); 7 genes represent the overlapping gene set. (**B**) Heatmap of differential gene expression of the 7 genes identified as both α3β1-dependent and LN332-responsive. (**C**) In total, 4 of the 7 genes identified in panel b displayed no significant difference upon LN332 treatment in MKα3− cells but showed significant upregulation with LN332 treatment in MKα3+ cells. *n* = 4; graphs are mean ± SEM; one-way ANOVA with multiple comparisons; ns, not significant; *, *p* < 0.05.

Since our in vivo data showed that LN332 treatment promoted integrin α3β1-dependent wound re-epithelialization (Figure 1), we aimed to develop an in vitro model to interrogate LN332-responsive changes to the keratinocyte transcriptome. We determined that treatment of MKα3+ cells with LN332-containing culture medium supported faster in vitro scratch wound closure (Figure S4), recapitulating our in vivo findings. Next, we treated MKα3− and MKα3+ cells with either LN332 or vehicle as control and sent the samples for RNA sequencing. Consistent with α3β1 being a regulator of the keratinocyte transcriptome [18], a large subset of MK genes (880) was α3β1-dependent. Since keratinocytes make and secrete endogenous LN332 [6,7], we expected that a rather small subset of genes would respond to treatment with exogenous LN332. Indeed, a small subset (19 genes) was found to be responsive to exogeneous LN332 treatment, and only 7 of these genes were also α3β1-dependent (Figure 3A,B), suggesting that the remaining 12 genes may involve other LN332 receptors (e.g., integrin α6β4). Four genes—Sox11, Nedd9, NGF, and Cavin3—exhibited a response to LN332 in MKα3+ cells, but not in MKα3− cells (Figure 3C). Interestingly, all four of these genes have been linked, directly or indirectly, to the regulation of cell migration/motility in different contexts [39,40,41,42,43].

### 3.3. Keratinocyte-Derived NGF Is an α3β1-Dependent, LN332-Responsive Factor That Promotes Keratinocyte Migration In Vitro

**Figure 4:**
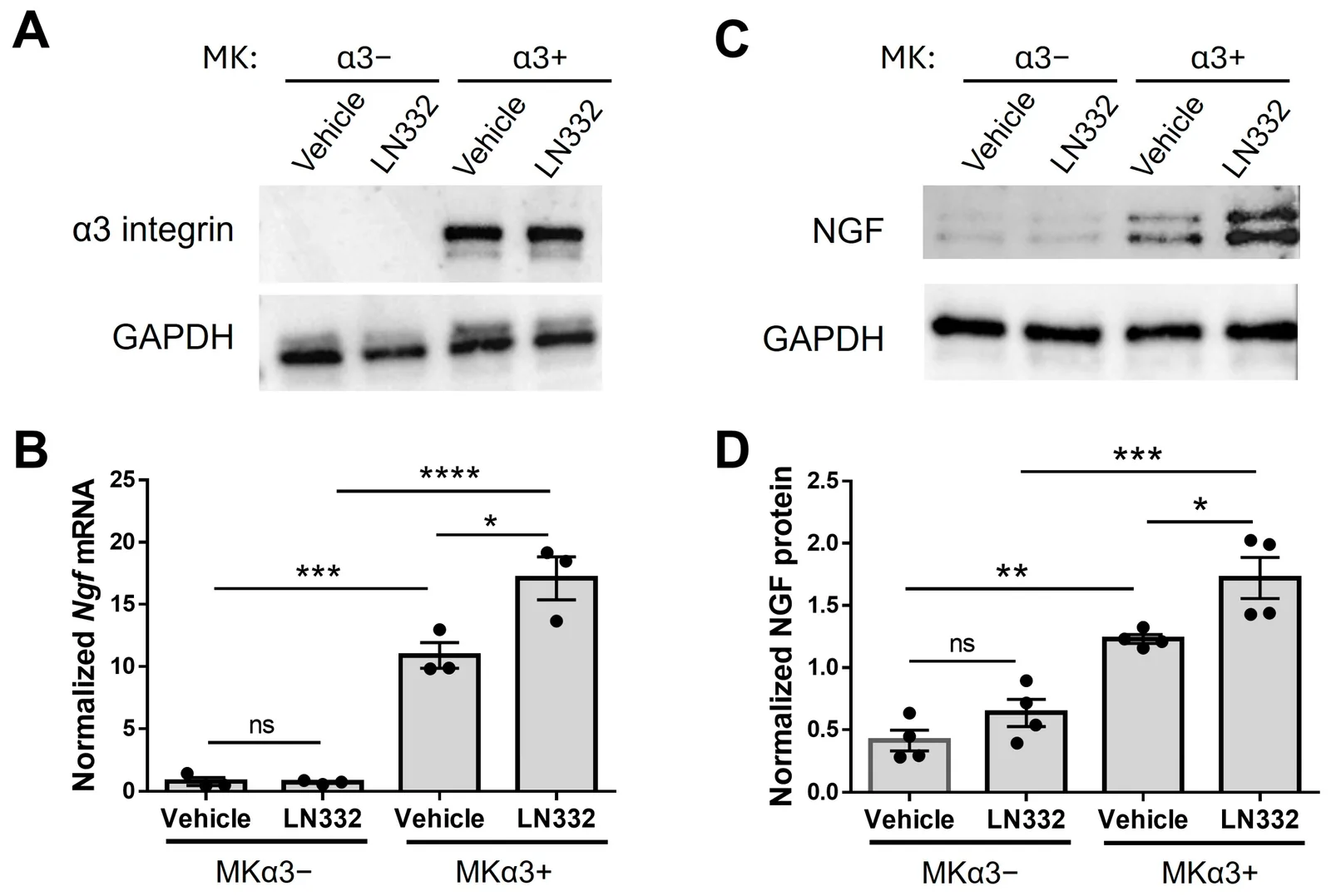
NGF is α3β1-dependent and LN332-responsive in keratinocytes. MKα3− and MKα3+ cells were treated with LN332 or vehicle control; samples were prepared for qPCR and immunoblotting. (**A**) Representative immunoblot confirms appropriate α3 integrin expression in MK cell lines; GAPDH, loading control. (**B**) Real-time qPCR measures *Ngf* mRNA (*n* = 3) and (**C**,**D**) immunoblotting measures NGF protein; (**C**) representative immunoblots shown; GAPDH, loading control. (**D**) Quantitation of NGF protein from immunoblots shown in (**C**); *n* = 4. (**B**,**D**) Graphs are mean ± SEM; one-way ANOVA with multiple comparisons; ns, not significant; *, *p* < 0.05; **, *p* < 0.01; ***, *p* < 0.001; ****, *p* < 0.0001.

NGF is of particular interest, since NGF levels are often reduced in non-healing wounds, and NGF treatment has been shown to increase the rate of wound closure in several wound models [44,45]. Consistent with the RNAseq data, quantitative real-time RT-PCR showed that *Ngf* mRNA levels are enhanced in MKα3+ cells compared to MKα3− cells, and that MKα3+ cells showed further *Ngf* mRNA enhancement with LN332 treatment, while MKα3− cells did not (Figure 4A,B). Consistently, a similar result was observed at the protein level by immunoblotting for NGF (Figure 4C,D).

**Figure 5:**
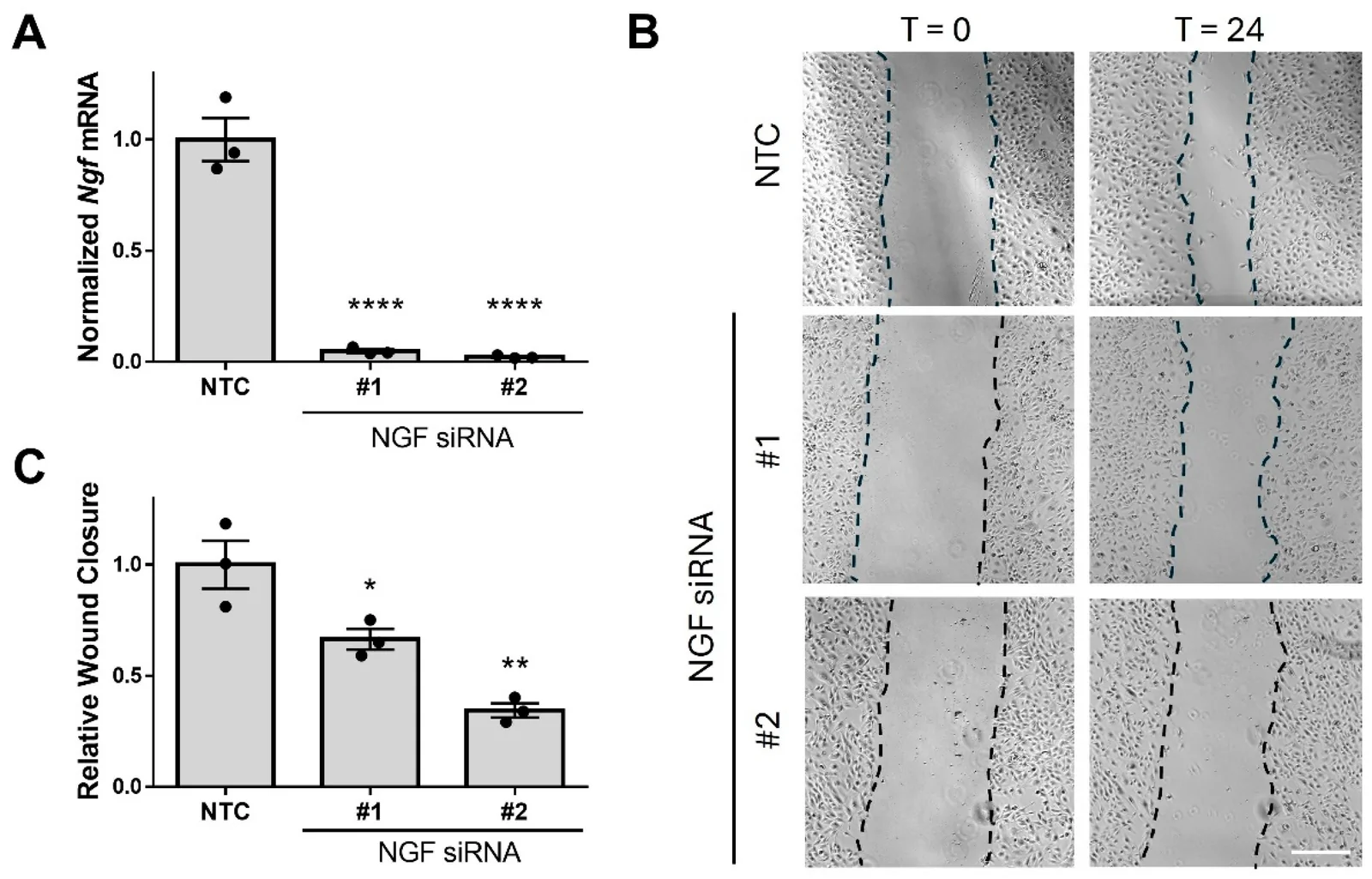
*Ngf* knockdown inhibits in vitro scratch wound closure. MKα3+ cells were transfected with *Ngf*-targeting siRNAs #1 or #2, or with a non-targeting control siRNA (NTC) for 72 h. (**A**) Real-time qPCR measures *Ngf* mRNA to confirm knockdown (*n* = 3). (**B**,**C**) In vitro scratch wound assays were performed and imaged at time 0 and 24 h. (**B**) Representative images from each timepoint are shown; dotted lines mark borders of the original (T = 0) and healing (T = 24) scratch wound; scale bar = 500 μm. (**C**) Graph shows relative wound closure. (**A**,**C**) Data are mean ± SEM; *n* = 3; one-way ANOVA with multiple comparisons; *, *p* < 0.05; **, *p* < 0.01; ****, *p* < 0.001.

Next, we used an siRNA approach to knockdown *Ngf* in MKα3+ cells, then performed a scratch wound assay to determine whether *Ngf* depletion reduces keratinocyte migration in vitro. Treatment of MKα3+ cells with *Ngf* siRNAs #1 and #2 significantly decreased the level of *Ngf* mRNA compared to a non-targeting control (NTC; Figure 5A). *Ngf* siRNA #1 and #2 yielded a 95% and 97% knockdown efficiency of *Ngf*, respectively, at the mRNA level (Figure 5A). Scratch wound closure over a 24 h period was significantly reduced in keratinocytes with *Ngf* knockdown compared to NTC (Figure 5B,C), indicating that NGF is required for efficient keratinocyte migration.

Treatment of keratinocytes with an activating ERK peptide was shown to promote keratinocyte migration in vitro and in a wound model in vivo [46]. Since ERK signaling is activated by integrin α3β1 in keratinocytes [47], and NGF has been shown to be regulated by ERK signaling in another context [48], we aimed to determine if NGF expression by keratinocytes is regulated by ERK signaling. Treatment of MKα3+ cells with the MEK/ERK inhibitor trametinib significantly reduced the level of pERK, indicating successful inhibition (Figure S5A,B). Concomitant with ERK inhibition, we observed reduced NGF level (Figure S5C,D), indicating that ERK signaling regulates NGF expression in keratinocytes, which is consistent with the LN332-integrin α3β1-ERK-NGF signaling axis that regulates keratinocyte migration.

### 3.4. Epidermal NGF Is α3β1-Dependent and LN332-Responsive in Murine Wounds In Vivo

**Figure 6:**
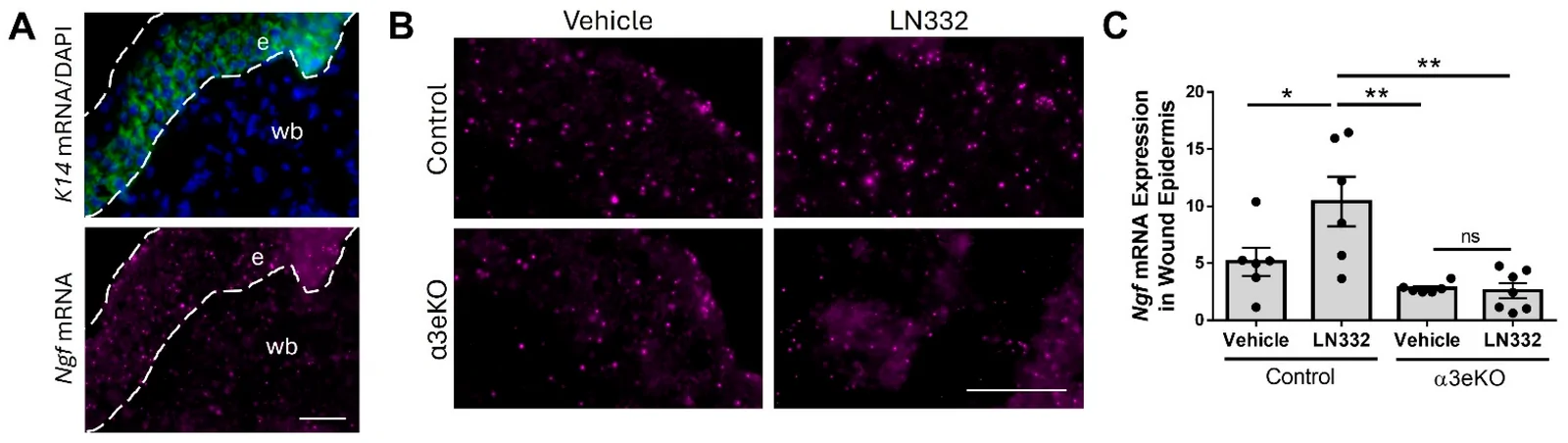
Induction of *Ngf* by LN332 is α3β1-dependent in wound keratinocytes in vivo. Cryosections of 3-day, partially re-epithelialized, LN332- and vehicle-treated wounds were prepared as in Figure 1. RNA ISH to detect *K14* mRNA (green, to mark epidermis; blue, DAPI) and *Ngf* mRNA (purple) was performed. (**A**) Representative images show ROI site selection, marking wound epidermis; e, epidermis; wb, wound bed; scale bar = 50 μm. (**B**) Representative images from all experimental groups show *Ngf* mRNA puncta fully within an area of wound epidermis; scale bar = 50 μm. (**C**) Graph shows quantitation of *Ngf* mRNA in wound epidermis; mean ± SEM; *n* ≥ 6 wounds per treatment group; one-way ANOVA with multiple comparisons; ns, not significant; *, *p* < 0.05; **, *p* < 0.01.

Finally, we aimed to evaluate whether NGF expression is α3β1-dependent and LN332-responsive in vivo by evaluating our murine wounds (described in Figure 1). Since several stromal cell types may contribute NGF to the wound microenvironment, we used RNAScope ISH, which allows for the visualization of single-molecule mRNA within tissue [49], to assess *Ngf* mRNA expression specifically within the wound epidermis (Figure 6A). Analysis of wound cryosections with a probe set specific for murine *Ngf* mRNA showed that this transcript was reduced substantially in the epidermis of α3eKO wounds compared with control wounds, and that LN332 treatment stimulated *Ngf* transcript above the level of vehicle treatment specifically in α3β1-expressing control wounds (Figure 6B,C). This finding shows that epidermis-derived NGF is α3β1-dependent and LN332-responsive in wounds in vivo. Overall, our findings indicate that LN332 treatment supports cutaneous wound closure in α3β1-competent epidermis, and that resulting gene expression changes in wound keratinocytes, such as the upregulation of the pro-healing factor NGF may in part underlie this effect.

## 4. Discussion

A deeper understanding of the mechanisms that support wound re-epithelialization is essential for developing strategies to accelerate wound closure and, in turn, prevent infection or the development of chronic wounds. During re-epithelialization, leading-edge keratinocytes deposit LN332 into the provisional BM, facilitating α3β1-dependent keratinocyte migration, and previous studies have shown that keratinocyte migration and polarization in vitro and wound re-epithelialization in vivo are regulated by integrin α3β1 [14,15,16,17]. Utilizing in vitro cell culture models and in vivo murine wound models, the current study finds that exogenous LN332 treatment supports keratinocyte migration and wound re-epithelialization, and that expression of integrin α3β1 by keratinocytes is required for this effect (Figure 1 and Figure 2). These findings demonstrate that topical treatment of wounds with full-length LN332 is sufficient to support re-epithelialization, likely via a LN332-α3β1 interaction that stimulates α3β1-dependent keratinocyte migration.

Additionally, our previous studies have shown that epidermal integrin α3β1 is a key regulator of the keratinocyte transcriptome and secretome [18,22]. Our current work shows that a small subset of the keratinocyte integrin α3β1-dependent transcriptome is also responsive to exogenous LN332 treatment (Figure 3) and may help support keratinocyte migration. It is important to note that keratinocytes make and secrete LN332 [6,7]. Therefore, the genes we call “LN332-responsive” are responding to treatment with additional recombinant LN332 and likely represent a small fraction of the genes that are regulated by the endogenous LN332-α3β1 axis. NGF, Sox11, Nedd9, and Cavin3 are the four genes that we identified as α3β1-dependent and LN332-responsive, and they have all been implicated in regulating cellular migration in different contexts [39,40,41,42,43]. Due to the previously described role for NGF in supporting wound healing [44,45], we validated this factor as α3β1-dependent and LN332-responsive in vitro and in vivo in the current study (Figure 4 and Figure 6). We further showed that NGF is required for efficient keratinocyte migration in an in vitro scratch wound assay (Figure 5). These data indicate that transcriptomic changes, such as upregulation of NGF, may further stimulate keratinocyte migration beyond a direct integrin-based migratory mechanism. However, future studies are needed to fully elucidate the biological importance of transcriptionally regulated elements in mediating keratinocyte migration and wound closure within the LN332–integrin α3β1 axis of epithelial repair.

An earlier study reported that topical LN332 treatment of murine diabetic wounds did not improve wound closure [50], possibly reflecting differences in diabetic versus non-diabetic wound healing or differences in the LN332 preparations used. For example, our studies utilized recombinant full-length LN332 while the previous study used LN332 derived from primary human foreskin keratinocytes [50]. The latter study ensured that the purified LN332 retained the necessary globular α3 laminin subunit domains required for binding to integrin α3β1. However, since full-length LN332 was not available at that time, the approach did not consider other potentially important domains, such as the cleavable γ2 laminin subunit domains required for incorporation into the epidermal BM [8]. Indeed, the authors of that study conceded that application of precursor LN332 may be necessary to improve wound healing [50], and concluded that future studies will be necessary to determine if stable incorporation of exogenous LN332 is required for its ability to stimulate integrin-dependent migration in vivo. Furthermore, the previous approach involved antibody conjugating the LN332 to a Tegaderm wound dressing [50], which may have further compromised the ability of LN332 to collect within the provisional wound matrix to stimulate integrin-dependent keratinocyte migration. More recently, however, a 12 amino acid sequence in the laminin α5 subunit globular domain, which is not present in LN332, was demonstrated to be sufficient to accelerate healing of murine diabetic wounds [51], suggesting that thoughtful laminin-inspired peptide development may also provide benefit and that full-length laminins may not be required in all cases.

Our current study demonstrates that LN332 supports acute wound closure, indicating potential utility of exogenously added LN332 as a wound therapy. While future studies are needed to determine the efficacy of full-length LN332 treatment in chronic or diabetic wound models, it is important to note the following studies that indicate the potential efficacy of LN332 treatment across wound pathologies. Gentamicin was shown to induce LN332 and improve wound healing in patients with junctional epidermolysis bullosa, a blistering skin disorder [52]. Moreover, reduced deposition of LN332 was observed in slow-healing irradiated skin in a rat model [53], indicating that aberrancies in LN332 deposition underlie some slow-to-heal wounds. These studies indicate that LN332-based wound therapies may have a broad relevance.

Other future studies should evaluate the impact of LN332 treatment on the wound healing process beyond re-epithelialization, including assessment of the quality and integrity of the nascent basement membrane, granulation tissue formation, collagen deposition, and wound inflammation. Interestingly, our earlier work has demonstrated that epidermal α3β1 promotes the wound macrophage population through the secreted factor CSF-1 [54]. It remains to be seen whether LN332 treatment stimulates other α3β1-dependent reparative processes beyond re-epithelialization, such as changes in the inflammatory response. Combining similar studies with a bacterial wound challenge, for instance, would also be informative towards understanding the complete utility of LN332 in the wound healing process.

An important consideration for the therapeutic translation of LN332 is its large, heterotrimeric structure. It is likely that recombinant LN332 must retain the appropriate heterotrimeric structure and functional domains to engage integrin α3β1 and for incorporation into the nascent BM. This may present challenges related to protein stability, delivery, and formulation in the wound environment. As a multidomain extracellular matrix protein composed of α3, β3, and γ2 chains, recombinant LN332 may be susceptible to proteolytic degradation within the protease-rich milieu of a wound, and maintaining the structural integrity and biological activity of the heterotrimer following topical administration will be essential. In addition, the relatively large size and complex assembly of LN332 may complicate production, purification, storage, and formulation compared with smaller recombinant proteins or peptides, although the commercial availability of recombinant LN332 indicates that these challenges are not insurmountable. Nevertheless, topical delivery offers a potentially practical route for localized administration, and strategies such as incorporation into protective biomaterials or hydrogel formulations could potentially enhance protein stability, retention, and sustained availability at the wound site. Importantly, the present findings provide proof-of-concept that augmenting the LN332–integrin α3β1 axis can promote re-epithelialization, but further studies will be necessary to determine the optimal dose, formulation, treatment frequency, and stability of recombinant LN332 in clinically relevant wound environments. Thus, although the complexity of LN332 presents translational challenges, its localized mechanism of action and the potential for topical delivery support further investigation of LN332-based approaches as a therapeutic strategy for impaired cutaneous wound healing.

Indeed, hydrogel- and ECM-based wound therapies have been gaining traction in recent years [55,56,57,58], and distinct ECM proteins, such as collagen I, have been shown to support aspects of wound healing and can be found in commercially available wound dressings [5]. We found that higher doses of collagen I trended toward enhancing wound re-epithelialization in our model (Figure S3). However, unlike collagen I, LN332 is not expected to contribute substantially to the tensile strength of the healing wound. Therefore, these ECM components may work best in combination in the wound clinic, providing complementary benefits by promoting both re-epithelialization and tissue integrity. Furthermore, the use of collagen I in therapeutic wound dressings indicates that treatment with other ECM proteins, like LN332, should be practically feasible.

## Data Availability

RNA sequencing data is available at the GEO repository, accession number GSE336489. Other data will be made available upon request.

## Supplementary Materials

The following supporting information can be downloaded at https://www.mdpi.com/article/10.3390/biomedicines14091969/s1, Figure S1. Confirmation of α3 integrin epidermal knockout in α3eKO mice. K14^CreERT^::α3^flx/flx^ mice were treated with topical 4OHT five and three days prior to wounding to knockout epidermal integrin α3β1, or with a vehicle control. Cryosections of skin were prepared and immunofluorescence with anti-α3 integrin was performed; representative images shown; e, epidermis; d, dermis; scale bar = 50 μm. Figure S2. The mRNA levels of all subunits of integrin α3β1 and LN332 are upregulated in wounds compared to intact skin. A publicly available dataset analyzing intact human skin versus wounded human skin (GSE28914) was interrogated for differential gene expression (DGE) of the following gene panel: *ITGA3*, *ITGB1*, *LAMA3*, *LAMB3*, and *LAMC2*. DGE is displayed as a heatmap showing expression relative to intact skin. Benjamini-Hochberg False Discovery Rate (FDR) adjusted *p*-values; **, *p* < 0.01; ***, *p* < 0.001. Figure S3. Assessment of collagen treatment in murine wound closure. Full-thickness wounds on mouse dorsum were treated for three consecutive days with 3.75 mg/mL collagen, 0.01 mg/mL collagen, or vehicle control. Cryosections of 3-day, partially re-epithelialized wounds were prepared. Immunofluorescence with anti-K14 to mark the epidermis was performed and wound gaps were measured. Graph shows wound gap measurements; data are mean ± SEM; *n* ≥ 6 wounds per treatment group; student’s *t*-test comparing each collagen treatment to vehicle; ns, not significant; *p* value approaching significance reported. Figure S4. Treatment of MKα3+ cells with LN332-containing culture medium supported faster in vitro scratch wound closure. In vitro scratch wound assays were performed in MKα3+ cells. After time 0 images were taken, cultures were treated with either LN332- or vehicle-containing culture medium (control) and placed in incubator to “heal” for 18 h. Scratch wounds were re-imaged after 18 h. (A) Representative images from each timepoint are shown; scale bar = 500 μm. (B) Graph shows % wound closure; data are mean ± SEM; *n* = 4; student’s *t*-test; *, *p* < 0.05. Figure S5. ERK signaling regulates NGF expression in keratinocytes. MKα3+ cells were treated with MEK/ERK inhibitor, trametinib, and cell lysates were prepared and immunoblotted for (A,B) pERK with ERK as a loading control and (C,D) NGF with GAPDH as loading control. (A,C) Representative blots shown. (B,D) Graphs show normalized levels of (B) pERK and (D) NGF relative to their respective loading controls; data are mean ± SEM; *n* = 3; student’s *t*-test; **, *p* < 0.01.
